# Meiotic Interactors of a Mitotic Gene *TAO3* Revealed by Functional Analysis of its Rare Variant

**DOI:** 10.1534/g3.116.029900

**Published:** 2016-06-14

**Authors:** Saumya Gupta, Aparna Radhakrishnan, Rachana Nitin, Pandu Raharja-Liu, Gen Lin, Lars M. Steinmetz, Julien Gagneur, Himanshu Sinha

**Affiliations:** * Department of Biological Sciences, Tata Institute of Fundamental Research, Mumbai 400005, India; † Gene Center, Ludwig-Maximilians-Universität, 81377 Munich, Germany; ‡ Genome Biology Unit, European Molecular Biology Laboratory, 69117 Heidelberg, Germany; § Department of Genetics, School of Medicine, Stanford University, California 94305; ** Stanford Genome Technology Center, Stanford University, Palo Alto, California 94304; †† Department of Informatics, Technische Universität München, 85748 Garching, Germany; ‡‡ Department of Biotechnology, Bhupat and Jyoti Mehta School of Biosciences, Indian Institute of Technology Madras, Chennai 600036, India; §§ Initiative for Biological Systems Engineering, Indian Institute of Technology Madras, Chennai 600036, India

**Keywords:** rare variant, *TAO3*, meiosis, transcriptome profiling, allelic variant

## Abstract

Studying the molecular consequences of rare genetic variants has the potential to identify novel and hitherto uncharacterized pathways causally contributing to phenotypic variation. Here, we characterize the functional consequences of a rare coding variant of *TAO3*, previously reported to contribute significantly to sporulation efficiency variation in *Saccharomyces cerevisiae*. During mitosis, the common *TAO3* allele interacts with *CBK1*—a conserved NDR kinase. Both *TAO3* and *CBK1* are components of the RAM signaling network that regulates cell separation and polarization during mitosis. We demonstrate that the role of the rare allele *TAO3(4477C)* in meiosis is distinct from its role in mitosis by being independent of *ACE2*—a RAM network target gene. By quantitatively measuring cell morphological dynamics, and expressing the *TAO3(4477C)* allele conditionally during sporulation, we show that *TAO3* has an early role in meiosis. This early role of *TAO3* coincides with entry of cells into meiotic division. Time-resolved transcriptome analyses during early sporulation identified regulators of carbon and lipid metabolic pathways as candidate mediators. We show experimentally that, during sporulation, the *TAO3(4477C)* allele interacts genetically with *ERT1* and *PIP2*, regulators of the tricarboxylic acid cycle and gluconeogenesis metabolic pathways, respectively. We thus uncover a meiotic functional role for *TAO3*, and identify *ERT1* and *PIP2* as novel regulators of sporulation efficiency. Our results demonstrate that studying the causal effects of genetic variation on the underlying molecular network has the potential to provide a more extensive understanding of the pathways driving a complex trait.

The ‘common disease, common variant’ rationale of genome-wide association studies (GWAS) is being challenged owing to the limited fraction of disease heritability explained by mapped common variants (Manolio *et al.* 2009; Zuk *et al.* 2014). Not considering the potential effects of rare variants has been suggested as one of the potential contributors to this ‘missing’ heritability (Saint Pierre and Génin 2014). This view has been substantiated by identification of rare variants carrying a considerable risk for autism, schizophrenia, and epilepsy (Stankiewicz and Lupski 2010). Thus, characterizing the functional role of rare variants associated with complex diseases has the potential to reveal new biology, and to provide opportunities for treatment (Cirulli and Goldstein 2010; Zuk *et al.* 2014). Although multiple variants for various diseases have been mapped, they have not been able to provide targets for treatment. This is because many variants have been mapped in noncoding regions of the genome, and we do not understand their functional role in disease development. Moreover, detailed characterization is required even for causal coding variants to fully understand their role in phenotypic variation. This necessitates the need to identify the mediating molecular pathways connecting a variant to the phenotype, which has the potential to greatly expand the set of possible targets for molecular intervention (Gagneur *et al.* 2013).

Yeast sporulation efficiency is a complex trait, and many polymorphisms contributing to this trait variation have been mapped in yeast strains from diverse ecological niches. These include sporulation genes such as *IME1*, an initiator of meiosis (Gerke *et al.* 2009), and *RIM15*, a glucose-sensing regulator of meiosis (Lorenz and Cohen 2014). In addition to several sporulation genes, coding polymorphisms were also identified in two genes, *MKT1*, a putative RNA-binding protein, and *TAO3*, a putative scaffolding protein (Deutschbauer and Davis 2005), both of which were uncharacterized for their role in meiosis. The high sporulating SK1 strain contains the causative nonsynonymous polymorphisms *MKT1 (89G)* and *TAO3 (4477C)*, while the low sporulating S288c strain contains *MKT1(89A)* and *TAO3(4477G)* (Deutschbauer and Davis 2005). In our previous work, we showed that the *MKT1(89G)* variant increased sporulation efficiency by interacting with regulators of mitochondrial retrograde signaling and nitrogen starvation during sporulation (Gupta *et al.* 2015). *TAO3* encodes a highly conserved scaffolding protein that is a component of the RAM (Regulation of Ace2p activity and cellular Morphogenesis) signaling network. In addition, Tao3 activates another RAM network protein, Cbk1—a NDR protein kinase (Du and Novick 2002; Hergovich *et al.* 2006). The RAM network, which consists of Cbk1, Hym1, Kic1, Mob2, Sog2, and Tao3 proteins, is involved in an Ace2-dependent cell separation and cellular progression during mitotic division (Nelson *et al.* 2003). Ace2, a transcription factor, peaks early in mitosis and is involved in G_1_/S transition (Spellman *et al.* 1998). The RAM network regulates cellular progression in a Ace2-independent manner as well (Bogomolnaya *et al.* 2006). While components of the RAM network interact with *TAO3* during mitosis, none of these interactions provide clues as to its role in the developmental processes of meiosis and sporulation.

Here, we characterized the functional role of *TAO3(4477C)* in sporulation efficiency variation by elucidating the molecular pathways linking this mitotic gene to meiosis. We compared phenotypes of a pair of S288c-background strains differing only in the causal *TAO3* polymorphism. By studying the genome-wide transcriptional dynamics of these strains during sporulation, we predicted *TAO3(4477C)*-associated candidate mediator genes. A genetic interaction assay between these candidate genes and *TAO3* alleles identified regulators of tricarboxylic acid cycle and gluconeogenic enzymes as causal and novel regulators of sporulation efficiency.

## Materials and Methods

### Yeast strains and media

The yeast strains were grown in standard conditions at 30° in YPD (1% yeast extract, 2% bacto peptone, 2% dextrose). Allele replacement strain YAD331 (Deutschbauer and Davis 2005) was a S288c-background diploid strain containing the homozygous causative sporulation polymorphism *TAO3(4477C)*. Whole-genome resequencing of YAD331 with S288c strain as the reference strain identified two additional polymorphisms (Supplemental Material, Figure S6 and Table S7). Three consecutive backcrosses were performed between the haploid derivative of YAD331 and the haploid reference strain (S288c) to remove these secondary polymorphisms. After the backcrosses, the sole genetic difference between the reference S288c strain and the backcrossed allele replacement strain was at the *TAO3(G4477C)* position, which was confirmed by performing PCR-based sequencing 650 bp up and downstream around the two secondary polymorphisms and the *TAO3* polymorphic nucleotide. This backcrossed strain was diplodized to make it homozygous at the *TAO3(4477C)* position, and was called “T strain” in this study; the diploid parental strain S288c was called “S strain”. All gene deletions in the study were made in haploids of the T and S strains, except for those made in strain SK1 (Table S8). Deletions were performed and verified as described previously (Goldstein and McCusker 1999; Gietz and Woods 2002). The haploid strains were diplodized using pHS2 plasmid (containing a functional *HO*) and mating types were confirmed by performing MAT PCR (Huxley *et al.* 1990). All experiments in this study were performed using the diplodized parent strains and their diploid derivatives. To replace the endogenous *TAO3* promoter (–150 to –1 bp upstream of the start site) in the T strain with a tetracycline-responsive promoter, a *tetO_7_*-based promoter substitution cassette containing *kanMX4* was amplified from the plasmid pCM225 (Bellí *et al.* 1998b). The diploid T strain with this *tetO_7_*-based cassette is termed P_Tet_-*TAO3(4477C)* strain. The primers for sequencing, deletions, and their confirmations are listed in Table S9.

### Phenotyping

**Figure 1:**
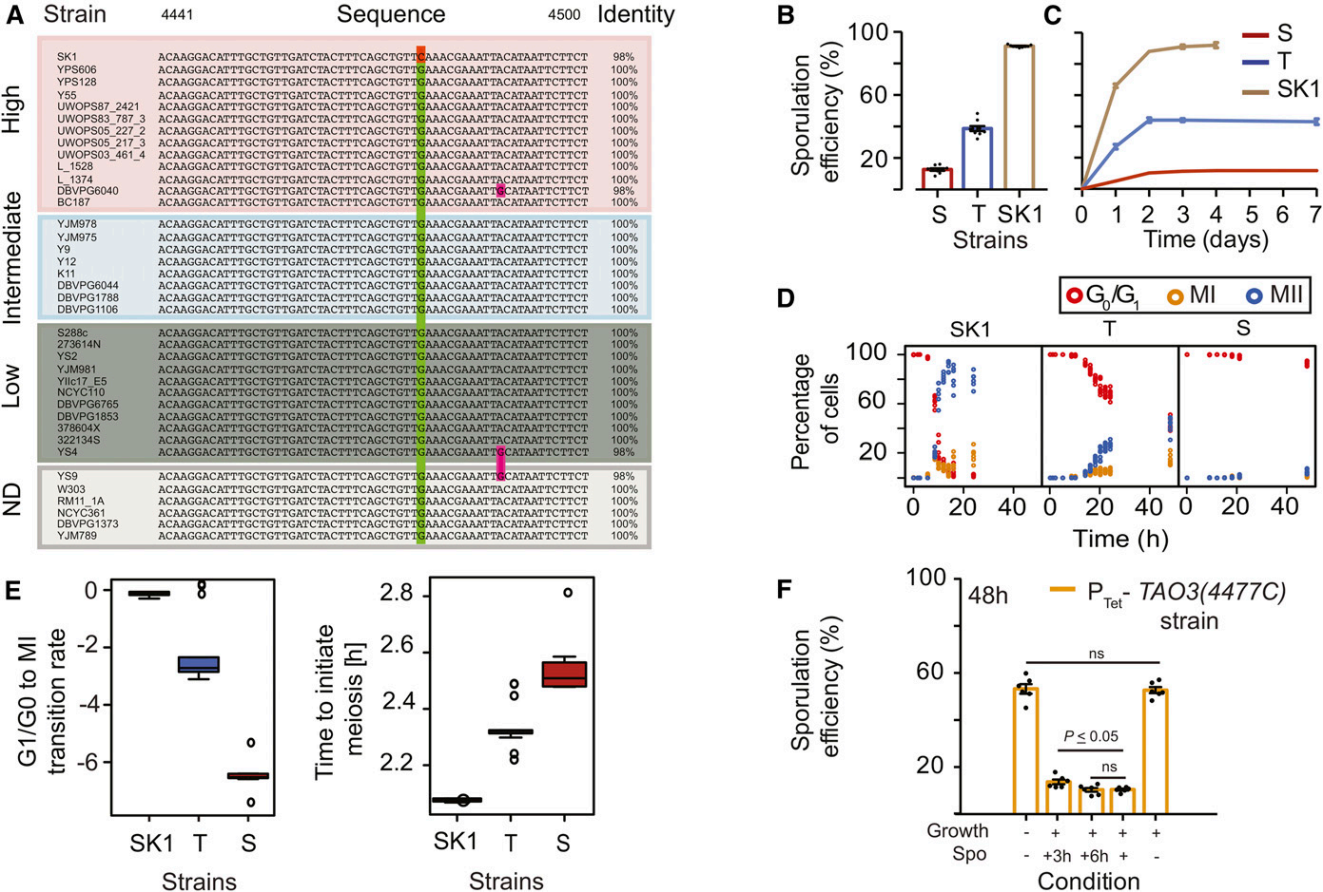
Role of *TAO3* in sporulation efficiency. (A) Comparison of genomic sequences of *TAO3* (4441–4500) across the SGRP collection (Liti *et al.* 2009). The 4477th position of *TAO3* consists of the sporulation causative variant where identical nucleotides are indicated by the same color. Identity indicates the percentage match between the nucleotides in the shown region of the gene. The strains are ordered according to their mean sporulation efficiency (Tomar *et al.* 2013): high (60–100%), intermediate (10–60%), low (0–10%) and ND (not determined). (B) Bar plots represents the mean sporulation efficiency after 48 hr of the SK1, T and S strains. The sporulation efficiency data are indicated as circles. (C) Line graphs represent the mean sporulation efficiency of the S, T, and SK1 strains measured until saturation, *i.e.*, until sporulation efficiency did not vary for three consecutive days. (D) Percentage of one-, two- and four-nuclei states of the T strain (*y*-axis) *vs.* time in sporulation medium (*x*-axis). One-nucleus stage is indicated as red circles (G_0_/G_1_ phase), two-nuclei state as yellow circles (completion of Meiosis I, MI phase), and blue circles is four-nuclei stage (completion of Meiosis II, MII phase). (E) Bootstrap distribution of the time to initiate meiosis and the rate of transition from G_1_/G_0_ into MI, estimated from time courses in (D). See *Materials and Methods* for details. (F) Conditional expression of *TAO3(4477C)* during sporulation in P_Tet_-*TAO3(4477C)* strain. The *y*-axis is the mean sporulation efficiency in 48 hr. No doxycycline in growth (YPD) or spo (YPA + sporulation) medium is depicted as “–” condition on the *x*-axis, and addition of doxycycline is depicted as “+” under those conditions. The “+3h” condition in Spo indicates that doxycycline was present throughout in the growth medium, and in the sporulation medium until 3 hr, after which cells were sporulated in the absence of doxycycline. The “+6h” condition indicates that doxycycline was present throughout in both the growth and sporulation medium until 6 hr, after which cells were sporulated in the absence of doxycycline. *P* values were calculated by an unpaired *t*-test. Error bars are SEM.

Sporulation efficiency estimation at 48 hr, progression through meiotic landmark events meiosis I (MI) and meiosis II (MII), and its quantitation was done as described previously (Gupta *et al.* 2015). For quantitation of meiotic landmarks in the T strain, parametric curves assuming delayed and 1st order kinetics were fitted to DAPI-stained meiotic progression time-course data, and fitting uncertainties were estimated by bootstrapping (File S1). Cell cycle progression data for S288c and SK1 strains was taken from Gupta *et al.* (2015) (Figure 1, D and E). Conditional expression of *TAO3(4477C)* was performed by constructing strain P_Tet_-*TAO3* (details in File S1), which was responsive to the tetracycline analog doxycycline (Bellí *et al.* 1998a, 1998b). Doxycycline (2 µg/ml) was added to growth and sporulation media to decrease expression of the *TAO3* gene. For each strain, a minimum of three biological replicates was used, and the experiment was carried out a minimum of two times; ∼300 cells were counted per replicate. Fold difference was calculated as the ratio of mean sporulation efficiencies of the two strains A and B when the sporulation efficiency of A is greater than that of B. Growth curve analysis was performed for individual strains grown in YPD in 96-well plates. Cells were grown overnight in YPD to saturation, reinoculated in YPD in transparent 96-well plates with a starting OD_600_ of 0.01, and grown with shaking at 30° for 24 hr in a Tecan Infinite M200 microplate reader. Doubling times were calculated from OD measurements of liquid cultures at a wavelength of 600 nm in the Tecan reader. For each strain, four technical replicates for each of the three biological replicates were used. Raw sporulation efficiency values are given in Table S10.

### Statistical test for calculating sporulation efficiency

To compare sporulation efficiency, two statistical tests were used: the pair test and the interaction test. The pair test tests the null hypothesis that the two given strains (S and T) have the same sporulation efficiency.

The number, $y_{i,k}$, of sporulated cells (four-nuclei count) among the total number of cells, $n_{i,k}$, of strain *i* in replicate experiment *k* was modeled with a quasi-binomial generalized linear model using the *logit* link function, and subject to a common log-odds ratio,$\beta_{i}$, between replicates, *i.e.*,:

$$\log\left(\frac{{\text{μ}}_{i,k}}{n_{i,k}-{\text{μ}}_{i,k}}\right)={\text{β}}_{i}$$

for all *k*, where ${\text{μ}}_{i,k}=E\left(y_{i,k}\right)$.

The pair test tests the null hypothesis of equality of log-odds ratios for two strains, *i* and *j*, *i.e.*, $H_{0}:{\text{β}}_{\text{i}}={\text{β}}_{\text{j}}$.

In the case of the S and T strains, the interaction test tests the null hypothesis that the effect of mutation A is independent of the effect of mutation B, taking strain T as the reference background. This test thus compares four strains: mutation A only, mutation B only, both A and B, and neither A nor B (T strain). Here, the S strain was considered as a T strain mutated for *TAO3(4477)*. For every interaction test, we considered the dataset of the four strains of interest, and fitted a quasi-binomial generalized linear model using the *logit* link function and subject to:

$$\log\left(\frac{{\text{μ}}_{i,k}}{n_{i,k}-{\text{μ}}_{i,k}}\right)={\text{β}}_{0}+{\text{β}}_{A}A_{i}+{\text{β}}_{B}B_{i}+{\text{β}}_{A,B}A_{i}B_{i}$$

for all *k*, where *A_i_* and *B_i_* are indicator variables of the mutations A and B in strain *i*, respectively. The interaction test tested the null hypothesis that the odds ratio of sporulation in the double mutant equals the product of the odds ratios of each mutation, *i.e.*, $H_{0}:{\text{β}}_{A,B}=0$.

Both the pair test and the interaction test were implemented in the statistical language R with the function *glm()* assuming a constant variance function fitted by maximizing the quasi-likelihood, and using the *t*-test on the tested parameters (Gupta *et al.* 2015).

### Whole genome gene-expression profiling

Sporulating yeast cell collection at 0 hr, 30 min, 45 min, 1 hr and 10 min, 1 hr and 40 min, 2 hr and 30 min, 3 hr and 50 m, 5 hr and 40 min, and 8 h and 30 min (logarithmic time-series), RNA isolation, and cDNA preparation were performed as previously described (Xu *et al.* 2009). Samples were hybridized to *S. cerevisiae* yeast tiling array (Affymetrix, Cat# 520055). Arrays at each time point for both strains were normalized to each other using the *vsn* normalization method (Huber *et al.* 2002). For qPCR, aliquots of cDNA were used in real-time PCR analyses with reagents from Kapa SYBR fast Universal qPCR master mix (Kapa Biosystems) in the Eppendorf Real-time PCR system according to manufacturer’s protocol. For each strain, four technical replicates for each of the three biological replicates were used. The primers used are given in Table S9.

### Whole genome gene-expression analysis

Within each strain, the log_2_ expression values obtained were smoothed using *locfit* at optimized bandwidth parameter *h* = 1.2 (Figure S7), and base transformed for each transcript by subtracting the expression value at each time point from the baseline value at time point *t* = 0 hr (*t*_0_, Table S11). This log_2_ fold change value with respect to *t*_0_ is described as “expression” throughout the manuscript. To identify genes showing temporal differential expression between the T and S strains (Table S1), the method implemented in EDGE software was used to calculate statistically significant changes in expression between the T and S strains over time (Storey *et al.* 2005). The differentially expressed genes were clustered according to their temporal expression patterns using the time abstraction clustering algorithm implemented in the TimeClust software (Magni *et al.* 2008, see File S1). Four major clusters were identified in each strain: Cluster I (early trend), Cluster II (increasing trend), Cluster III (late trend), Cluster IV (repressing trend) (Table S2). The transcription factors regulating a cluster of genes were extracted using the YEASTRACT database (Teixeira *et al.* 2013). Only those transcription factors whose target genes were significantly enriched in the corresponding cluster were considered as candidate genes (*P* ≤ 0.05, odds ratio ≥ 1.5). The YEASTRACT database was also used in this study to obtain the regulation matrix of yeast for identifying target genes of regulators such as *UME6*. Target genes for *ACE2* were obtained from Nelson *et al.* (2003). Significantly enriched gene ontology (GO) terms by biological process (Bonferroni corrected *P* < 0.05, Table 1) were obtained from SGD Yeastmine (Balakrishnan *et al.* 2012).

**Table 1:** Functional GO categories of clusters in the T and S strains

Cluster	Functional GO Category	Genes
Early in T strain (Cluster I)	Carbohydrate metabolic process	*DOG1*, *YPI1*
	Ion transport	*AVT4*, *DAL5*
	Mitochondrion organization	*PPE1*, *UPS3*
	Cellular respiration	*COX5B*
Early in T strain (Cluster I) repressed in S strain (Cluster IV)	Carbohydrate metabolic process	*ALG6*, *DEP1*, *DOG1*, *TPS3*, *YPI1*
	Mitochondrial organization	*ATG33*, *COX20*, *PPE1*, *UPS3*

Comparison of functional GO categories of differentially expressed genes in the T strain clusters with the S strain. See Table S2 for the full list of genes in each cluster.

### Data availability

The array data for the T strain has been deposited in ArrayExpress (http://www.ebi.ac.uk/arrayexpress/) with accession number E-MTAB-3889. The entire genome sequence data for the T strain has been deposited in the European Nucleotide Archive (http://www.ebi.ac.uk/ena/) with the accession number PRJEB8698. The array data and the whole genome sequence data for the S strain were downloaded from Gupta *et al.* (2015). *TAO3* gene sequence data for *Saccharomyces* Genome Resequencing Project (SGRP) strains (Liti *et al.* 2009) was downloaded from (http://www.moseslab.csb.utoronto.ca/sgrp/). An additional 24 *TAO3* sequences were downloaded from the *Saccharomyces* Genome Database (SGD, http://www.yeastgenome.org/cgi-bin/FUNGI/alignment.pl?locus=YIL129C, date accessed: March 1, 2016). Detailed methods are described in File S1. Table S1 lists all differentially expressed genes between the T and S strains with their *P* and *Q* values calculated using EDGE. Table S2 lists genes in each cluster using TimeClust. Table S3 lists transcription factors regulating unique early (Cluster I) genes of the T strain. Table S4 lists transcription factors regulating unique increasing (Cluster II) genes of the T strain. Table S5 lists differentially expressed target genes of regulators of candidate genes mediating the affect of *TAO3*. Table S6 lists transcription factors regulating unique repressing (Cluster IV) genes of the S strain. Whole genome resequencing results for the *TAO3* allele replacement strain are described in Table S7. All the strains used in the study are listed in Table S8, which are available upon request. All the primers used in the study are listed in Table S9. The raw sporulation efficiency values of the strains are given in Table S10. Table S11 contains smoothed expression data, base transformed with respect to t_0_ for the T and S strains. Figure S1 shows mathematical modeling to identify stages of meiosis affected by *TAO3* causal allele. Figure S2 shows growth phenotype and *TAO3* expression in P_Tet_-*TAO3**(4477C)* strain. Figure S3 shows comparison of global gene expression between the T and S strains at t = 0h. Figure S4 shows comparison of genes showing early (Cluster I) and increasing trend (Cluster II) between the T and S strains. Figure S5 shows genes having early expression in the T strain show expression at later time points or repressed in the S strain. Figure S6 shows whole genome resequencing of *TAO3* allele replacement strain (YAD331, (Deutschbauer and Davis 2005) in comparison to the S288c reference strain. Figure S7 shows smoothing of normalized temporal data using *locfit*.

## Results

### Role of causative allele of TAO3 in sporulation efficiency variation

Analysis of *TAO3* nucleotide sequence of 38 *S. cerevisiae* strains in the SGRP database (Liti *et al.* 2009), and 24 strains in SGD, showed that the *TAO3(4477C)* allele of strain SK1 was a rare variant (minor allele frequency = 1.6%, Figure 1A). Deutschbauer and Davis (2005) mapped *TAO3(4477C)* as one of the causal alleles contributing to high sporulation efficiency while studying the genetic basis of phenotypic variation between S288c (low sporulating) and SK1 (high sporulating) strains. By introducing *TAO3(4477G)* in the S288c background, they constructed the allele replacement strain YAD331, which differed from the parental S288c strain [*TAO3(4477G)*] only for this variant. They also showed that YAD331 showed significantly higher sporulation efficiency than S288c in 48 hr. For this study, we confirmed by sequencing the presence of the *TAO3(4477G)* allele in the YAD331 strain, but also identified and removed several background mutations present in this strain (*Materials and Methods*). This cleaned version of the YAD331 allele replacement strain was called “T strain”. Sporulation analysis of the T strain reconfirmed that it sporulated threefold more efficiently at 48 hr in comparison to the S288c strain (“S strain”, *P* = 1.8 × 10^−10^, pair test in *Materials and Methods*, Figure 1B). Moreover, this fold-difference between the two strains remained constant even after a week in sporulation medium (Figure 1C). Studying the progression of meiotic phases showed that the T strain initiated meiosis within 12 hr (Figure 1, D and E). Quantitative comparison of the ‘time to initiate meiosis’, and the ‘rate of transition from G_1_/G_0_ into Meiosis I stage’, showed significant differences between the T and S strains (Figure 1, D and E, and Figure S1). This suggested that *TAO3(4477C)* affected entry of the T strain cells into initiating meiosis within 12 hr during sporulation. To further resolve when, during these 12 hr in the cells entering meiosis, *TAO3(4477C)* plays a functional role, this allele was placed under a tetracycline-responsive promoter [P_Tet_-*TAO3(4477C)* strain, see *Materials and Methods*]. In the absence of the tetracycline analog doxycycline, higher expression of *TAO3(4477C)* was observed in strain P_Tet_-*TAO3(4477C)* compared to its expression in the S strain (*Materials and Methods*, Figure S2). Addition of doxycycline significantly reduced *TAO3* expression, making it equivalent to the S strain (*Materials and Methods*, Figure S2). Concomitantly, sporulation efficiency of the P_Tet_-*TAO3(4477C)* strain was high in the absence of doxycycline, being equivalent to the S strain in the presence of doxycycline (Figure 1F). This suggested that high *TAO3(4477C)* expression was required for the high sporulation efficiency phenotype. We next reduced *TAO3(4477C)* expression only for 3 hr and 6 hr in sporulation medium by sporulating the P_Tet_-*TAO3(4477C)* strain in the presence of doxycycline for these specific time-periods. Sporulation efficiency of the P_Tet_-*TAO3(4477C)* strain was equivalent to the S strain whether *TAO3* expression was reduced for 48 hr or only for the first 6 hr during sporulation (Figure 1F). Reduction of *TAO3* expression for the first 3 hr only did reduce the sporulation efficiency of P_Tet_-*TAO3(4477C)* strain, but it was not equivalent to S strain (*P* = 0.02, Figure 1F). This showed that the *TAO3(4477C)* allele played a functional role in sporulation within the first 6 hr.

### Role of TAO3 in meiosis is distinct from its role during mitosis

**Figure 2:**
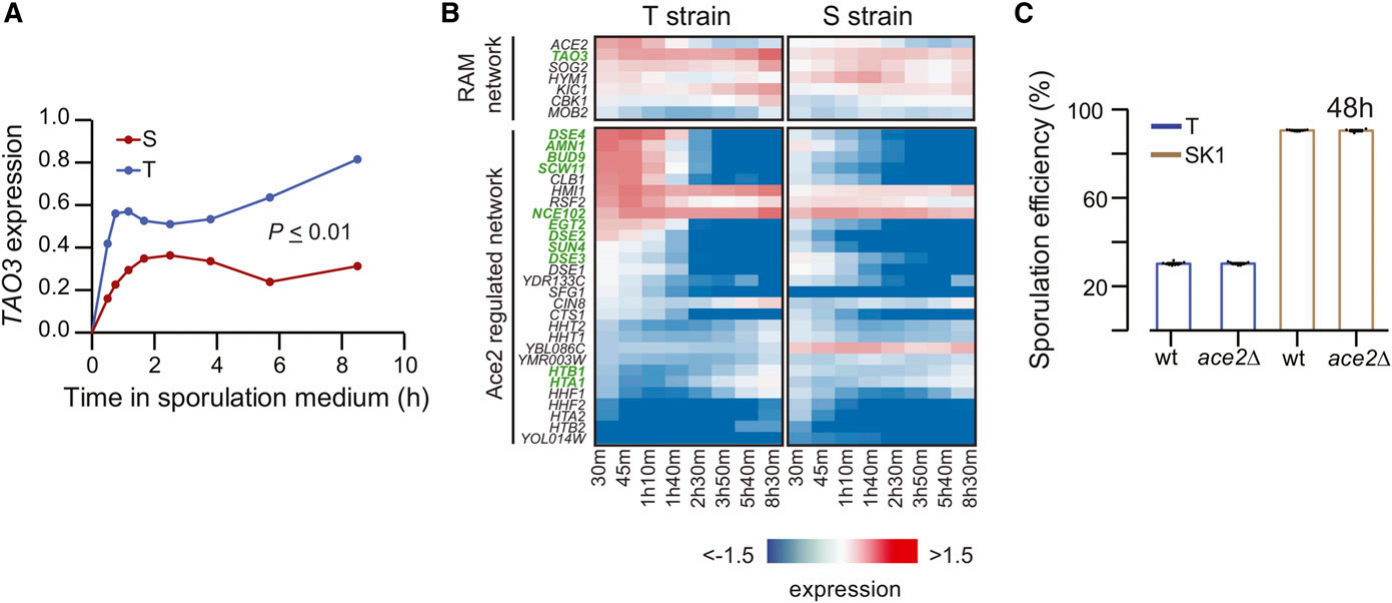
The role of *TAO3* in meiosis is distinct from its role during mitosis. (A) Expression profile (log_2_ fold change t_0_) of *TAO3* is given in the *y*-axis for the T (purple) and S strains (red), and the *x*-axis denotes the time in sporulation medium (data in Table S1 and Table S11). (B) Heatmap showing gene expression of RAM network genes and Ace2-regulated genes in the T and S strains. Gene names in green show differential expression (data in Table S1 and Table S11). (C) Bar plots represent the mean sporulation efficiency after 48 hr of the SK1 and T wild type (wt) and *ace2*∆ deletion strains. Pair and interaction tests (*Materials and Methods*) were performed to test significance.

Varying the gene expression of *TAO3(4477C)* affected the sporulation efficiency phenotype. Hence, to identify the molecular pathways affected by this causative allele, we studied the global gene expression dynamics during sporulation in the allele replacement strains. Time-resolved transcriptomes of the T and S strains were compared from 0 hr to 8 hr, 30 min in sporulation medium (*Materials and Methods*). At the initial time point (*t* = 0 hr), only 190 out of 6960 transcripts (∼3%) showed differential expression, with an enrichment for a single GO term, iron ion homeostasis (*P* = 0.04, post Holm-Bonferroni corrected, Figure S3). In contrast 1122 transcripts (including noncoding stable unannotated transcripts, Table S1) showed statistically significant differences in gene expression dynamics as a function of time between the two strains (false discovery rate cut-off 10%, when controlling for expression at *t* = 0 hr) . While *TAO3* was among the transcripts showing differential expression dynamics during sporulation (*P* = 0.004), none of its mitotic interactors were differentially expressed (Figure 2, A and B). Instead, we identified 11 *ACE2*-regulated genes (shown in green in Figure 2B) showing differential expression, and so we studied the effect of *ace2*∆ in the T strain and high sporulating SK1 strain. *ACE2* is known to regulate the budding phenotype (Voth *et al.* 2005), and we recapitulated the clumping phenotype of *ace2*∆ in both the T and SK1 strains. However *ace2*∆ did not affect sporulation efficiency of either the T or the SK1 strain (Figure 2C). An Ace2-independent effect of the RAM network on cellular polarization has been observed previously (Nelson *et al.* 2003); therefore, it is possible that this network could still be involved in meiosis.

To determine whether the mitotic interactors of *TAO3* could be distinct from its meiotic interactors, we again used P_Tet_-*TAO3(4477C)* strain and reduced *TAO3* expression only during the mitotic growth phase, *i.e.*, in glucose rich (YPD) medium. We observed no growth difference between P_Tet_-*TAO3(4477C)* strain with or without doxycycline and the T strain (Figure S2). Moreover, reduction of *TAO3* expression during growth had no effect on the high sporulation efficiency of P_Tet_-*TAO3(4477C)* strain (Figure 1F). These results suggested that *TAO3(4477C)* allele could have distinct meiosis-associated interactors that could explain its functional role during sporulation.

### Temporal gene expression profiling predicts TAO3(4477C)-specific interactors during sporulation

**Figure 3:**
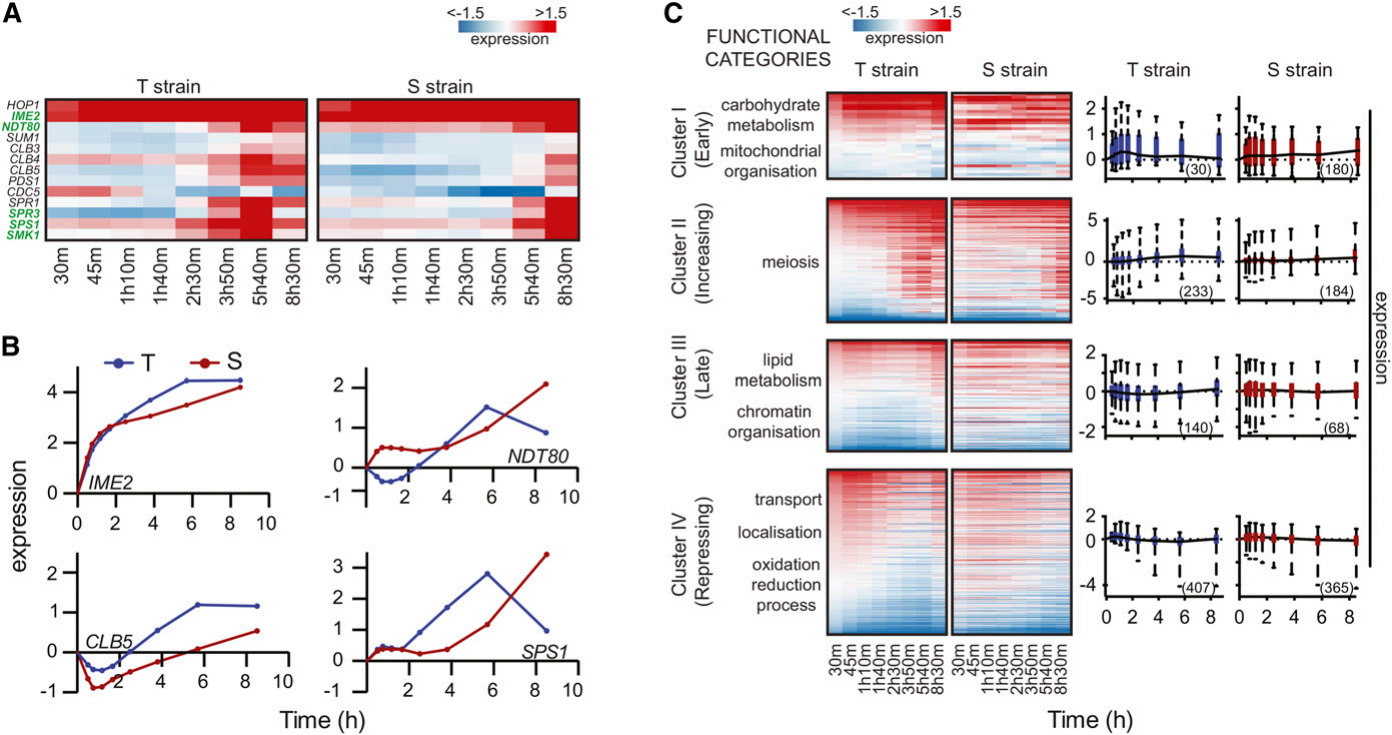
Global gene expression variation in presence of causative *TAO3* allele. (A) Temporal heatmap of meiotic genes in the T and S strains. The gene names shown in green are differentially expressed in the presence of *TAO3(4477C)*. (B) The expression profile (log_2_ fold change *t*_0_) for the meiotic landmark genes is given in the *y*-axis, and the *x*-axis denotes the time in sporulation medium. The red line represents the expression profile of the respective gene in the S strain, and the blue line is the same in the T strain. (C) Heatmap of the T and S strains showing differentially expressed genes across time, clustered according to expression profiles in T strain. Each row represents a single gene, and columns are time points of each strain (for gene list in each cluster, see Table S2). The order of genes is based on clustering of the T strain. Both the heatmap and boxplot consists of genes in each cluster of the T strain, and represent their expression profiles in both T and S strains. Functional GO categories of genes in each cluster of T strain are shown on left. The boxplots on the right represent the average expression profile of each cluster in the T, and the same genes in S strains. The number of genes in each cluster in a strain is indicated in brackets.

Since we observed the functional role of *TAO3(4477C)* within the first 6 hr of sporulation (Figure 1F), we investigated the differentially expressed genes showing an early and increasing trend in their expression profiles uniquely in the T strain. These genes were identified by comparing the clustered differentially expressed genes separately for T and S strains (*Materials and Methods*). Various sporulation genes, including crucial regulators of meiosis, namely *IME1*, *IME2*, *DMC1*, and *NDT80*, were enriched (*P* = 5.5 × 10^−12^) in a cluster showing increasing expression trend (Cluster II) during sporulation in the T strain (Figure 3B, *Materials and Methods*); ∼50% of Cluster II genes of the T strain showed a similar increasing trend in the S strain, including *IME1*, *IME2*, *DMC1*, *ECM11*, and *NDT80* (Figure S4 and Table S2). Interestingly, very few early-expressing genes (Cluster I) of the T strain overlapped with the S strain (7%, Figure S4). These genes belonged to biological processes that regulated entry into sporulation, such as carbohydrate metabolic process, ion transport, mitochondrial organization, and cellular respiration (Table 1). Furthermore genes involved in biological processes like carbohydrate metabolism and mitochondrial organization showed repression in the S strain (Table 1 and Figure S5). Therefore, to study the early effects of the causal *TAO3* allele, we identified regulators of only those differentially expressed genes that showed early and increasing expression uniquely in the T strain (Table S3 and Table S4).

**Figure 4:**
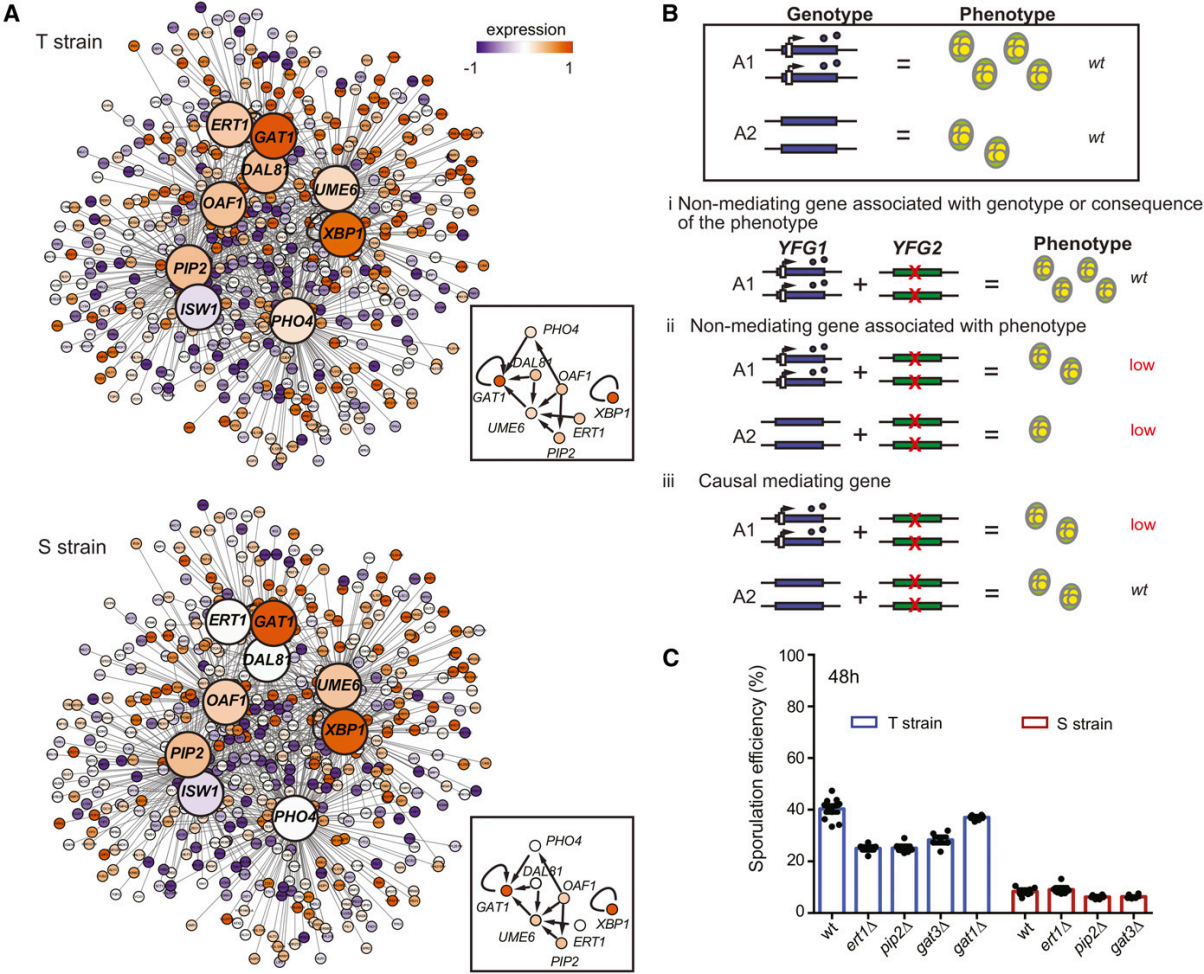
Identifying candidate genes mediating the allele-specific effects of *TAO3* during sporulation using the temporal gene expression data. (A) Regulatory network of candidate genes predicted to mediate the effects of *TAO3(4477C)* in sporulation. The candidate mediating genes are shown as bigger nodes (large circles), with their target genes (small circles) connected to them as straight lines. The box contains the protein network interactions of the candidate genes with the core sporulation gene *UME6*, obtained from YEASTRACT (*Materials and Methods*). Colors inside the nodes were calculated as an average of the first six time points in sporulation (early phase). For a complete list of interacting genes and their expression values, see Table S5 and Table S11, respectively. (B) Genetic model for functional validation of allele-specific interactors mediating sporulation efficiency variation. The wild type effect comparison of the two alleles A1 and A2 of the *YFG1* gene is shown inside the box. A1 is associated with high sporulation efficiency (wild type genotype and phenotype shown), and A2 is associated with low sporulation efficiency (wild type genotype and phenotype shown). Genetic interaction of these *YFG1* alleles with candidate mediating genes (*YFG2*) is represented: (i) representation of nonmediating gene associated with genotype only, or is a consequence of the phenotype, since *yfg2*∆ in the presence of A1 does not affect the wild type phenotype of A1; (ii) representation of nonmediating gene associated with the phenotype independent of the allele, since *yfg2*∆ in the presence of both A1 and A2 lowers (low) the phenotype; (iii) representation of causal mediating gene since *yfg2*∆ only in presence of allele A1 lowers the phenotype, and in the presence of allele A2 does not change the wild type phenotype of A2. (C) Bar plots represent the mean sporulation efficiency after 48 hr of the T and S wild type (wt), and the *ert1*∆, *pip2*∆, and *gat3*∆ strains. Pair and interaction tests (*Materials and Methods*) were performed to test significance. For the T strain, *gat1*∆ was nonsignificant, but *ert1*∆ (*P* = 2.1 × 10^−12^), *pip2*∆ (*P* = 6.1 × 10^−13^), and *gat3*∆ (*P* = 9.6 × 10^−10^) significantly reduced the mean sporulation efficiency. Significant interaction terms were obtained between the genetic backgrounds (S and T) and *ert1*∆ (*P* = 2.3 × 10^−4^) and *pip2*∆ (*P* = 0.04).

Regulators of unique genes in Cluster I and II of the T strain were enriched in nutrient metabolism and chromatin modification. These biological processes are important for initiation of meiosis (Neiman 2011). A core sporulation gene, *UME6*, which, together with *IME1*, induces expression of early meiotic genes (Kassir *et al.* 2003), thereby regulating pathways that initiate meiosis (Table 2; Lardenois *et al.* 2015), was also identified. Interestingly, along with *UME6*, we also identified 25 upstream regulators of *UME6* (Figure 4A, Table S3, Table S4, and Table S5), such as *ERT1*, *OAF1-PIP2*, and *DAL81*. *ERT1*, a regulator of carbon source utilization (Turcotte *et al.* 2010), is involved in the switch from fermentation to respiration in glucose-limiting conditions (Gasmi *et al.* 2014). *OAF1-PIP2* is a protein complex regulating lipid metabolism (Karpichev and Small 1998). *DAL81* is a regulator of the nitrogen-degradation pathway (Marzluf 1997). Interestingly, like *UME6*, *OAF1* target genes were repressed in the S strain (Cluster IV, Table S6). Earlier work in S288c and SK1 strains had shown upregulation of *ERT1*, *PIP2*, and *DAL81* in SK1 strain during sporulation (Primig *et al.* 2000). However, their deletion in S288c strain had no effect on sporulation efficiency (Deutschbauer *et al.* 2002). A few other interesting regulators that we identified, not upstream *UME6* (Table S3 and Table S4), included *GAT1*, a regulator of nitrogen metabolism (Ljungdahl and Daignan-Fornier 2012) and *GAT3*, a regulator of spore wall assembly (Lin *et al.* 2013). We next tested if these metabolic regulators interacted genetically with *TAO3(4477C)* during sporulation.

**Table 2:** Functional GO categories of regulators of clusters in the T and S strains

Functional GO Category	Regulators	*P* Value
Carbon metabolism	*ERT1*, *OAF1*, *PIP2*, *MIG1*, *MIG2*	1.9 × 10^−6^
Nitrogen catabolite regulation	*DAL81*, *DAL82*, *GAT1*, *UME6*	1.7 × 10^−5^
Chromatin modification	*ISW1*, *PHO2*, *PHO4*, *UME6*, *OAF1*, *XBP1*, *SIF2*, *RSC2*	1.4 × 10^−5^

Functional GO classification of the regulators of the differentially expressed genes showing early and increasing expression only in the T strain. See Table S3 and Table S4 for the full list of genes.

### Allele-specific functional validation identifies TAO3(4477C)-specific genetic interactors during sporulation

The candidate genes predicted from the above analysis could be either causal, mediating genes interacting with *TAO3(4477C)* during sporulation, or nonmediating consequential genes associated with only the genotype or only the phenotype. To identify the causal mediating genes, we used a genetic model described previously (Figure 4B; Gupta *et al.* 2015). According to this model, if a gene is associated only with the *TAO3* genotype, and not with the sporulation phenotype, or is expressed as a consequence of the phenotype, its deletion would not affect the T strain phenotype. On the contrary, if a gene had an independent role in the sporulation phenotype, irrespective of the *TAO3* genotype, its deletion will result in both a reduction in phenotype, and an additive effect, irrespective of the genetic background. Any significant deviation from this expectation would imply dependence on the genotype, with epistasis being an extreme case. In this scenario, deleting the candidate gene in T strain would affect the phenotype, while deleting the same gene in the S strain would not have an effect on the phenotype, making it a causal mediating gene. We used this model on the regulators we had identified as candidate genes. While *gat1*∆ had no effect on sporulation efficiency of the T strain, *ert1*∆, *pip2*∆, and *gat3*∆ significantly reduced the mean sporulation efficiency of the T strain by about 1.5-fold (*P* = 2.1 × 10^−12^, *P* = 6.1 × 10^−13^, *P* = 9.6 × 10^−10^, respectively, pair test in *Materials and Methods*, Figure 4C). Significant interaction terms were obtained between the genetic backgrounds (S and T), and *ert1*∆ and *pip2*∆ (*P* = 2.3 × 10^−4^, *P* = 0.04, *Materials and Methods*) but not for *gat3*∆. This showed that the effect of *ert1*∆ and *pip2*∆ on sporulation efficiency was specific to *TAO3(4477C)*, making them causal mediating genes. *GAT1* and *GAT3* were nonmediating genes, the former could be associated with genotype only, or could be a sporulation-consequential gene, and the latter affected sporulation independently of the genotype. Therefore, the genetic model aided identification of true causal genes, namely *ERT1* and *PIP2*, which mediate the effect of the *TAO3* allelic variant on sporulation efficiency.

## Discussion

Strong effects on phenotypic variation have been observed as a consequence of rare coding variants (Cohen *et al.* 2004, 2005). Tao3 is conserved from yeast to humans (Hergovich *et al.* 2006), and its common allele has been functionally annotated solely for mitotic cell division (Du and Novick 2002; Nelson *et al.* 2003). Hence, it was surprising when the rare *TAO3* variant was mapped for sporulation efficiency variation (Deutschbauer and Davis 2005). In this study, using time-resolved transcriptome analysis, and an allele-specific genetic interaction assay, we identified *ERT1* and *PIP2* as the *TAO3(4477C)*-dependent mediators contributing to efficient meiosis. These genetic interactors of *TAO3(4477C)* are distinct from the mitotic interactors of *TAO3(4477G)*. In this study, we identified their novel regulatory role in sporulation efficiency.

During sporulation, the sole nonfermentable carbon source, such as acetate, becomes internalized into the tricarboxylic acid (TCA) and glyoxylate cycles. Gluconeogenesis utilizes TCA cycle intermediates, and synthesizes storage carbohydrates like trehalose that are utilized during late sporulation processes (Ray and Ye 2013). Hence, TCA, glyoxylate, and gluconeogenesis are the metabolic processes that are crucial for sporulation to progress. Reduced flux through any of these metabolic pathways is capable of reducing yeast sporulation efficiency (Aon *et al.* 1996). Genes encoding the crucial enzymes of these metabolic processes, such as *PFK1*, *CIT1*, and *CIT2*, are essential for sporulation (Deutschbauer *et al.* 2002). *ERT1* and *PIP2* are transcription factors that regulate these metabolic enzymes (Baumgartner *et al.* 1999; Gasmi *et al.* 2014). Taken together, our results show that the *TAO3(4477C)* allele interacts genetically with regulators of the TCA cycle and gluconeogenic enzymes during sporulation. The presence of the sporulation-associated polymorphism could modulate this interaction, thereby modulating the metabolic flux during early sporulation that could result in sporulation efficiency variation.

*IME1* acts as a bottleneck for the sporulation decision pathway. Lorenz and Cohen (2014) observed many natural sporulation-associated polymorphisms in genes that interacted with this input–output gene *IME1*, such as *RIM15*, a nutrient-sensing regulator of *IME2*. While *TAO3* and *MKT1* (Gupta *et al.* 2015) do not directly regulate *IME1*, in this study we show that variants in these two genes regulate early upstream metabolic processes that impinge on *IME1*. This provides support for the hypothesis that genes surrounding the signal transduction bottlenecks are reservoirs for accumulating causal genetic variants.

Tao3 localizes to polarized bud sites during mitosis (Nelson *et al.* 2003). Further determination of colocalization of *TAO3(4477C)* with membrane-associated *ERT1* and beta-oxidation regulators *OAF1-PIP2* can provide interesting clues of its function during sporulation. Similar to other scaffolding proteins like *Fry* (*Drosophila*) and *SAX-2* (*Caenorhabditis* *elegans*), Tao3 has multiple conserved Armadillo-like repeats (Hergovich *et al.* 2006), and the causal sporulation variant resides in one of them. Tao3(1493E) physically interacts with the RAM network proteins in rich growth conditions. It would be interesting to determine binding partners of causal Tao3(1493Q) during sporulation, and to study how the polymorphism affects the binding of this putative scaffolding protein. Additionally, a few iron metabolism genes were differentially expressed during growth phase prior to incubation in the sporulation medium (*t* = 0 hr). It would be interesting to study whether this metabolic effect of *TAO3* also plays a role in sporulation.

Even if the basic cellular network of an organism is known, it is crucial to understand how natural genetic variation and stress conditions modulate the molecular interactions within this network, resulting in differences in phenotypic outcomes (Gasch *et al.* 2016). This study highlights how the molecular interaction of *TAO3* variant with metabolic genes causes different phenotypic outcomes. Performing such functional studies following GWAS and linkage analysis could provide a deeper understanding of how causal genetic variants function at a molecular level. This understanding is crucial, especially in the field of personalized medicine, to make more reliable predictions regarding the functional consequences of an individual’s genotype on disease predisposition and treatment (Burga and Lehner 2013).

## Supplementary Material

Supplemental Material
