# Supplementary material for: Meiotic Interactors of a Mitotic Gene TAO3 Revealed by Functional Analysis of its Rare Variant
Source: G3 (Bethesda). 2016 Jun 14;6(8):2255–63. doi: 10.1534/g3.116.029900 (PMC4978881; doi:10.1534/g3.116.029900)
Supplement: Supplemental Material [file supp_6_8_2255__index.html]

Meiotic Interactors of a Mitotic Gene TAO3 Revealed by Functional Analysis of its Rare Variant — Supplemental Material 

# Meiotic Interactors of a Mitotic Gene *TAO3* Revealed by Functional Analysis of its Rare Variant

## Supplemental Material for Gupta *et al.*, 2016

**Files in this Data Supplement:**

- Figure S1 - Mathematical modeling to identify stage of meiosis affected by *TAO3* causal allele. (.pdf, 78 KB)
- Figure S2 - Effect of doxycycline on growth and expression of *TAO3(4477C)*. (.pdf, 73 KB)
- Figure S3 - Comparison of global gene expression between the T and S strains at time t = 0h. (.pdf, 122 KB)
- Figure S4 - Comparison of genes showing increasing trend (Cluster II) and early (Cluster I) between T and S strains. (.pdf, 37 KB)
- Figure S5 - Genes showing early expression in T strain, show expression in later time points or are repressed in S strain. (.pdf, 59 KB)
- Figure S6 - Whole genome-resequencing of *TAO3* allele replacement strain (YAD331, (Deutschbauer and Davis 2005) in comparison to S288c reference strain. (.pdf, 86 KB)
- Figure S7 - Smoothing of normalized temporal data using *locfit*. (.pdf, 75 KB)
- File S1 - Detailed methods. (.pdf, 351 KB)
- Table S1 - Differentially expressed genes between T and S strains, with their P and Q values calculated using EDGE. (.xls, 134 KB)
- Table S2 - Differentially expressed genes between T and S strains, with their P and Q values calculated using EDGE. (.xls, 90 KB)
- Table S3 - Transcription factors regulating unique early (Cluster I) genes of the T strain. (.xls, 42 KB)
- Table S4 - Transcription factors regulating unique increasing (Cluster II) genes of the T strain. (.xls, 43 KB)
- Table S5 - Differentially expressed target genes of regulators of candidate genes mediating the affect of TAO3. (.xls, 79 KB)
- Table S6 - Transcription factors regulating unique repressing (Cluster IV) genes of the S strain. (.xls, 38 KB)
- Table S7 - Whole genome requencing results for the TAO3 allele replacement strain. (.xls, 35 KB)
- Table S8 - Strain names. (.xls, 35 KB)
- Table S9 - Primer names. (.xls, 37 KB)
- Table S10 - Raw sporulation efficiency data. (.xls, 42 KB)
- Table S11 - Smoothed expression data, base transformed with respect to t0 for T and S strains. (.xls, 2 MB)
