# Supplementary material for: Meiotic Interactors of a Mitotic Gene TAO3 Revealed by Functional Analysis of its Rare Variant
Source: G3 (Bethesda). 2016 Jun 14;6(8):2255–63. doi: 10.1534/g3.116.029900 (PMC4978881; doi:10.1534/g3.116.029900)
Supplement: Supplemental Material [file supp_g3.116.029900_FigureS1.pdf]

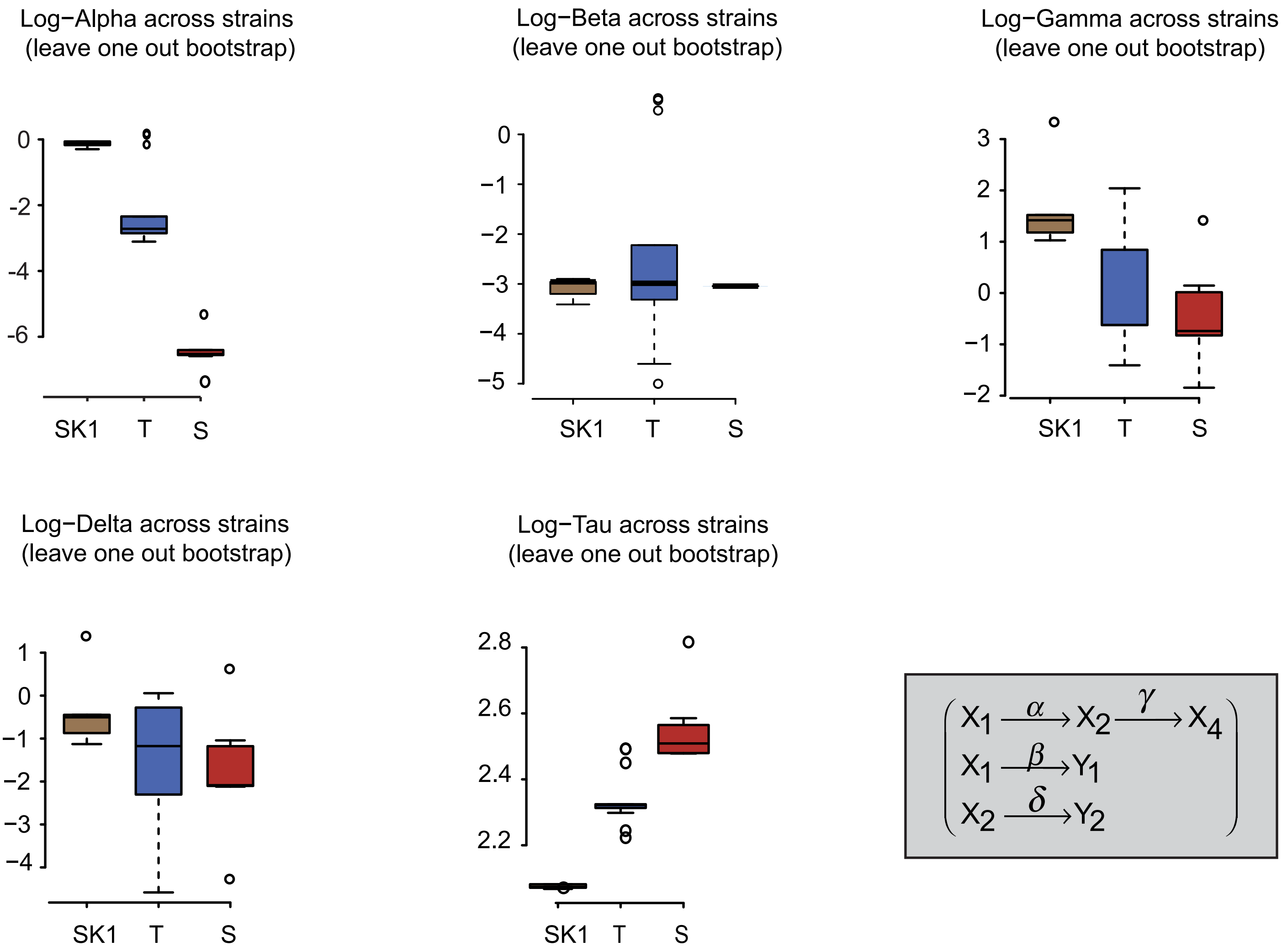

Figure S1. Mathematical modeling to identify stage of meiosis affected by *TAO3* causal allele. Log of parameters (y-axis) calculated according to the equation shown by studying the proportion of yeast cells entering various stages durig meiosis in SK1, T and S strain (x-axis). See methods for details. In the equation shown above the figure, X_1 is proportion of cells in 1-nucleus active stage, X_2 in 2-nuclei active stage, X_4 in 4-nuclei active stage, Y_1 is proportion of cells in 1-nucleus inactive stage, Y_2 in 2-nucleus inactive stage.
