# Supplementary material for: Meiotic Interactors of a Mitotic Gene TAO3 Revealed by Functional Analysis of its Rare Variant
Source: G3 (Bethesda). 2016 Jun 14;6(8):2255–63. doi: 10.1534/g3.116.029900 (PMC4978881; doi:10.1534/g3.116.029900)
Supplement: Supplemental Material [file supp_g3.116.029900_FigureS2.pdf]

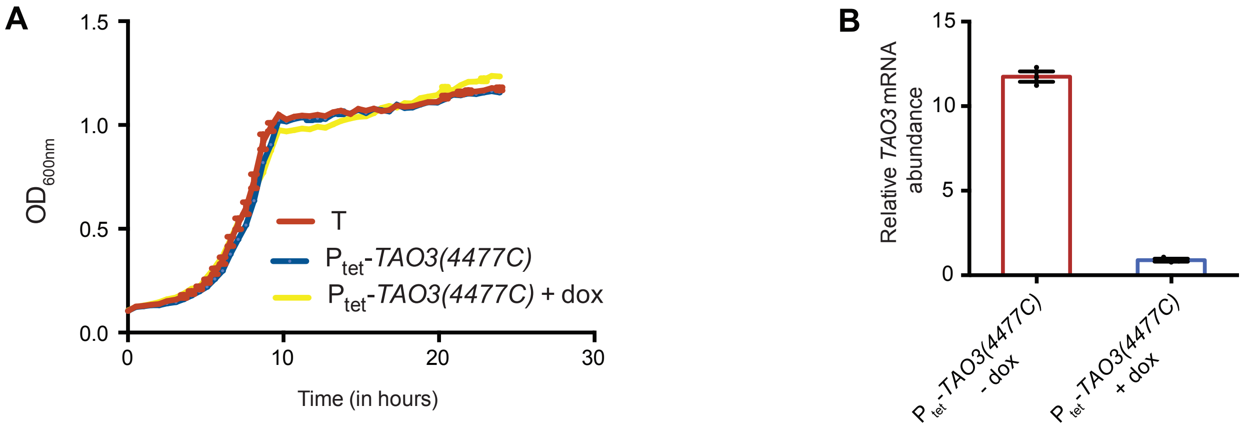

Figure S2. Effect of doxycycline on growth and expression of *TAO3(4477C)* (A) Growth curve of T strain in rich medium (red), P_{tet}-*TAO3(4477C)* strain in rich medium (blue) and P_{tet}-*TAO3(4477C)* strain in rich medium with doxycycline (yellow). X-axis shows the time for which the strain was growth and Y-axis depicts the mean absorbance at wavelength of 600nm (N=3, n=4). Error bars depict standard deviation. (B) Quantitation of *TAO3* mRNA abundance by performing quantitative PCR for P_{tet}-*TAO3(4477C)* strain in rich medium in absence and presence of doxycycline, relative to S strain is shown on the Y-axis (N=3, n=4). Error bars depict standard error of means.
