# Supplementary material for: Meiotic Interactors of a Mitotic Gene TAO3 Revealed by Functional Analysis of its Rare Variant
Source: G3 (Bethesda). 2016 Jun 14;6(8):2255–63. doi: 10.1534/g3.116.029900 (PMC4978881; doi:10.1534/g3.116.029900)
Supplement: Supplemental Material [file supp_g3.116.029900_FigureS3.pdf]

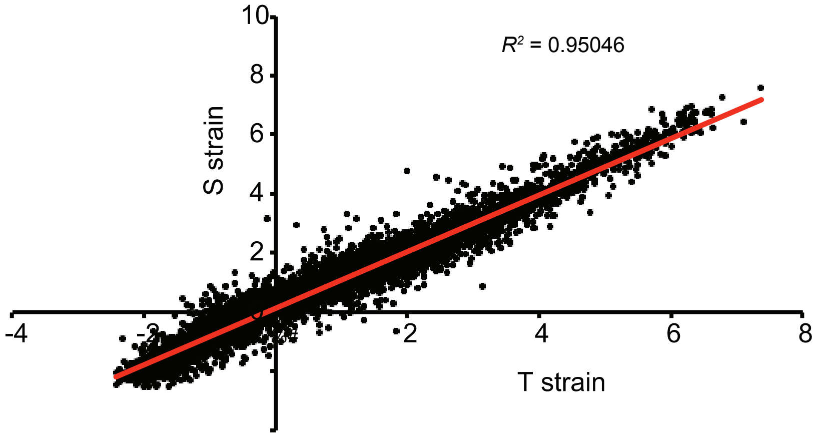

Figure S3. Comparison of global gene expression between the T and S strains at time t = 0h. Log_2 gene expression for all genes depicted as black dots is shown for t=0h for T strain on X-axis and S strain on Y-axis. Red line depicts the correlation between the two strains, which is 0.95.
