# Supplementary material for: Meiotic Interactors of a Mitotic Gene TAO3 Revealed by Functional Analysis of its Rare Variant
Source: G3 (Bethesda). 2016 Jun 14;6(8):2255–63. doi: 10.1534/g3.116.029900 (PMC4978881; doi:10.1534/g3.116.029900)
Supplement: Supplemental Material [file supp_g3.116.029900_FigureS4.pdf]

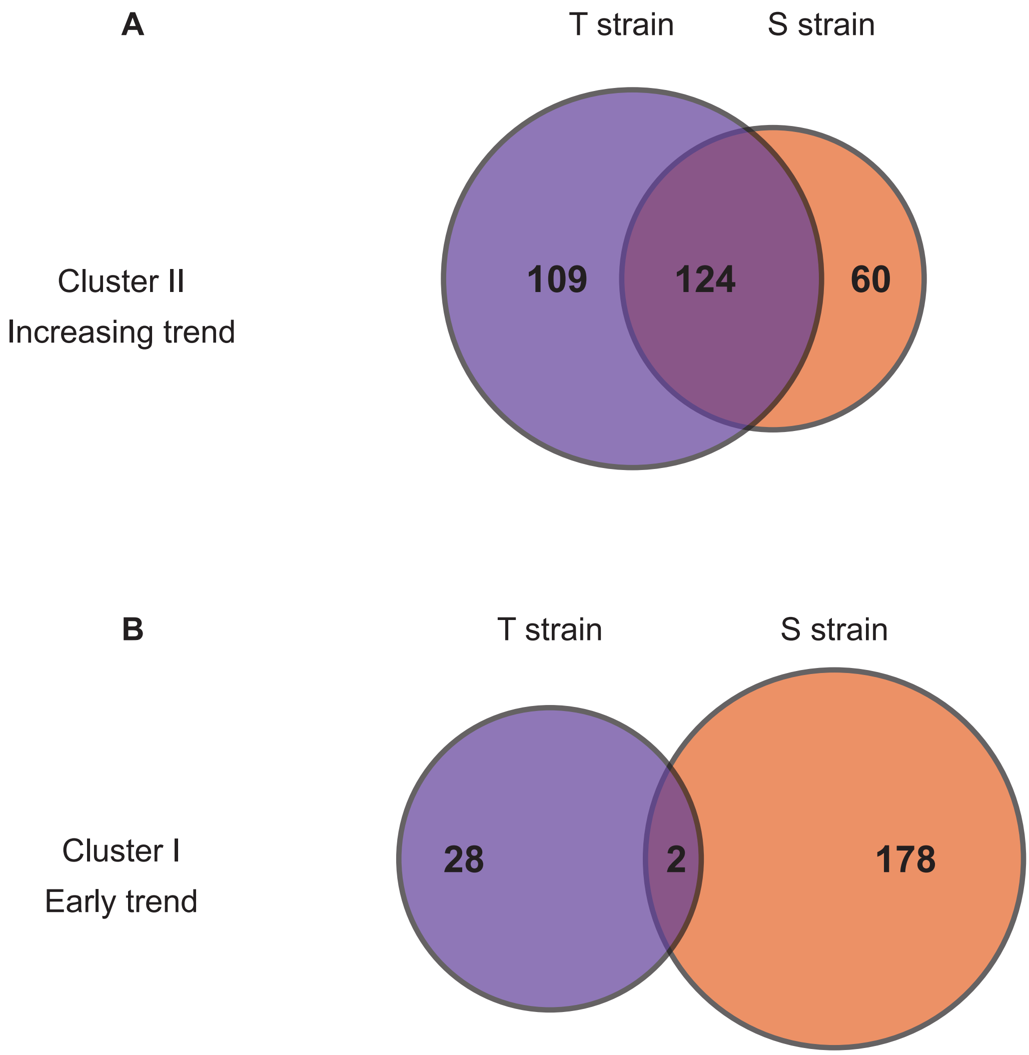

Figure S4. Comparison of genes showing increasing trend (Cluster II) and early (Cluster I) between T and S strains
