# Supplementary material for: Meiotic Interactors of a Mitotic Gene TAO3 Revealed by Functional Analysis of its Rare Variant
Source: G3 (Bethesda). 2016 Jun 14;6(8):2255–63. doi: 10.1534/g3.116.029900 (PMC4978881; doi:10.1534/g3.116.029900)
Supplement: Supplemental Material [file supp_g3.116.029900_FigureS5.pdf]

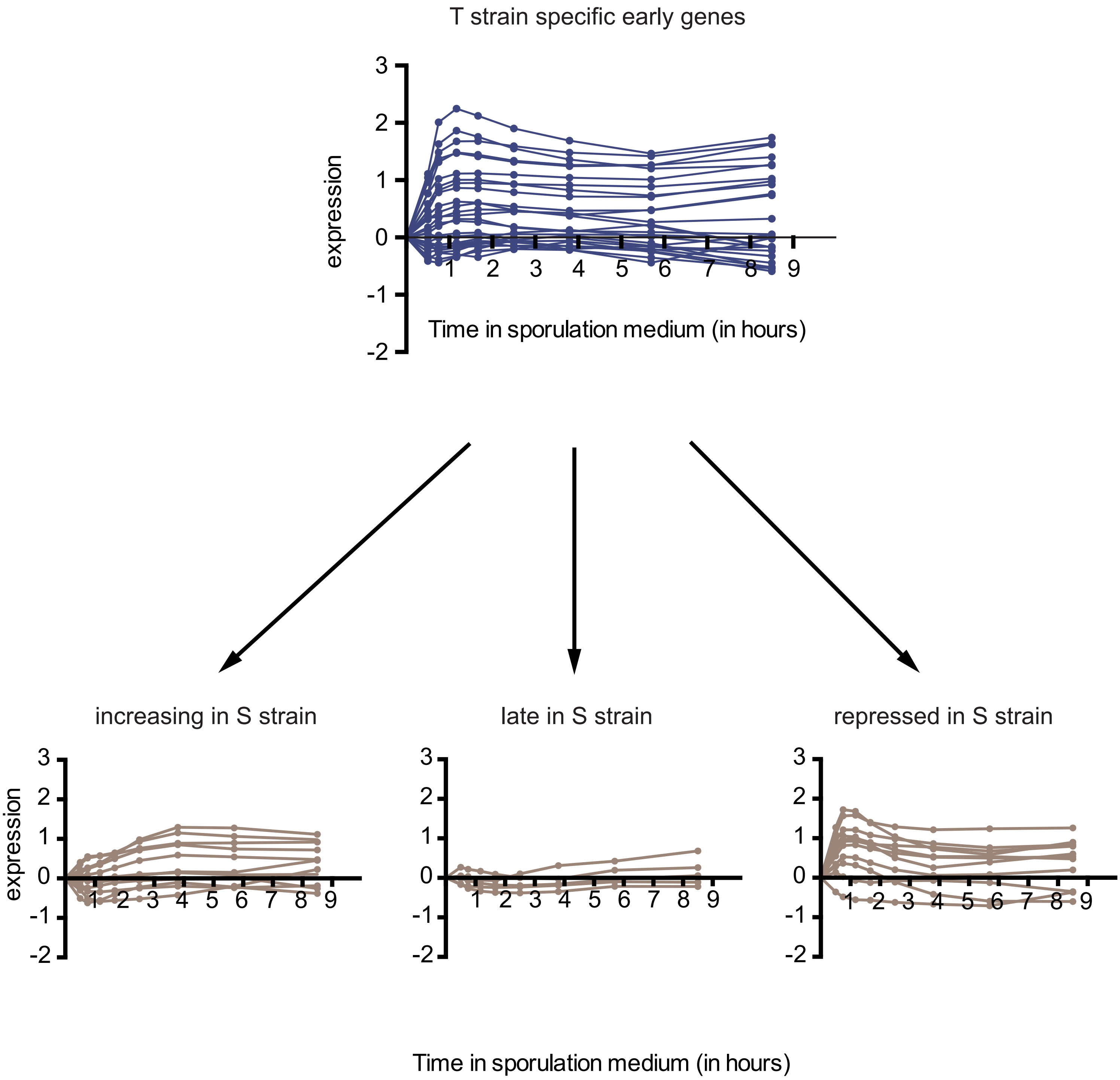

Figure S5. Genes showing early expression in T strain, show expression in later time points or are repressed in S strain. Gene expression is shown on Y-axis and time in sporulation medium is shown in X-axis. For gene list in each cluster see Supporting Table S6.
