# Supplementary material for: Meiotic Interactors of a Mitotic Gene TAO3 Revealed by Functional Analysis of its Rare Variant
Source: G3 (Bethesda). 2016 Jun 14;6(8):2255–63. doi: 10.1534/g3.116.029900 (PMC4978881; doi:10.1534/g3.116.029900)
Supplement: Supplemental Material [file supp_g3.116.029900_FigureS6.pdf]

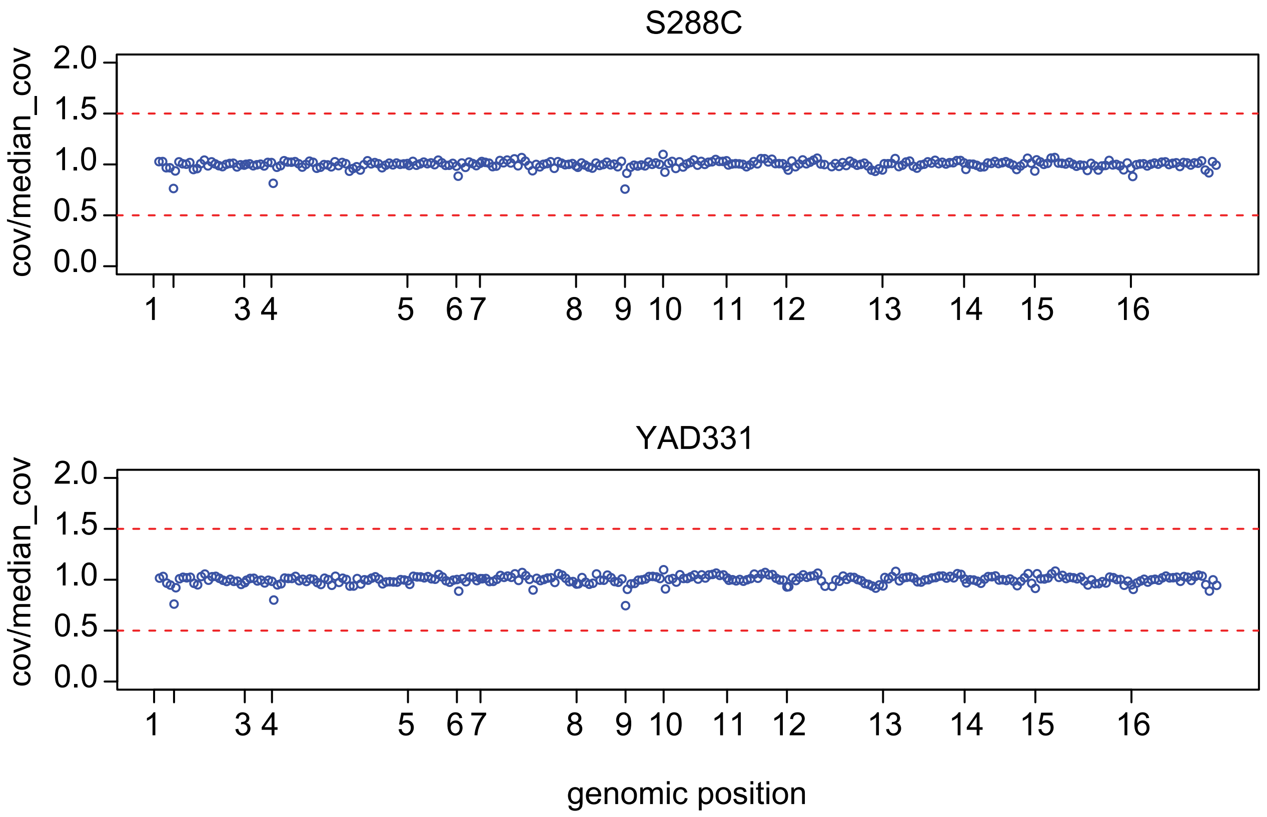

Figure S6. Whole genome-resequencing of *TAO3* allele replacement strain (YAD331, (Deutschbauer and Davis 2005) in comparison to S288c reference strain. Segmentation plots where each chromosome is divided into bins (shown as blue dots) depicted on the x-axis. Copy number of each bin relative to S288c reference strain is shown on the y-axis. Red dotted lines show the position where a chromosomal region (bin) would appear if there is a duplication (above 1.5) or deletion (below 0.5).
