# Supplementary material for: Meiotic Interactors of a Mitotic Gene TAO3 Revealed by Functional Analysis of its Rare Variant
Source: G3 (Bethesda). 2016 Jun 14;6(8):2255–63. doi: 10.1534/g3.116.029900 (PMC4978881; doi:10.1534/g3.116.029900)
Supplement: Supplemental Material [file supp_g3.116.029900_FigureS7.pdf]

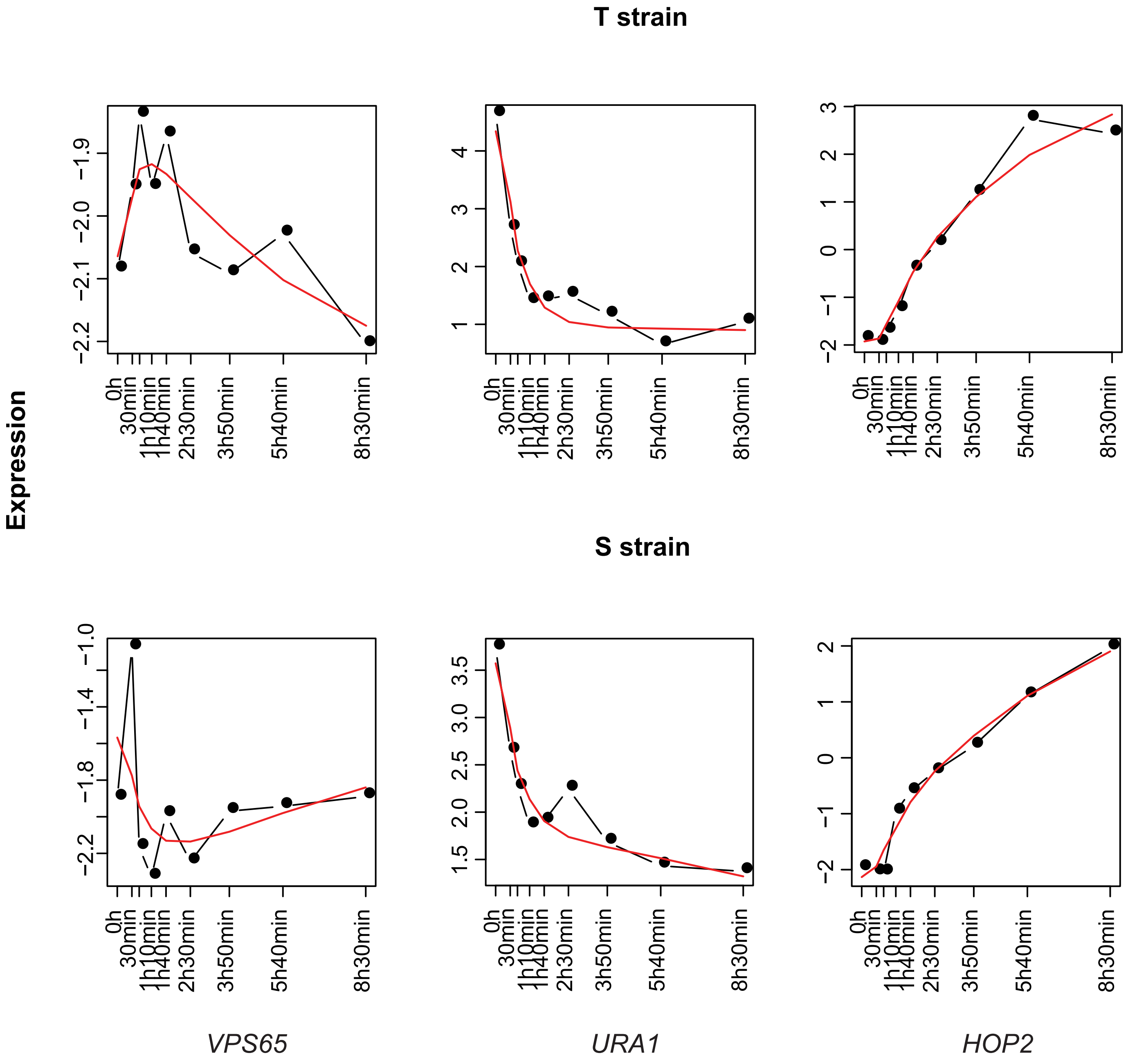

Figure S7. Smoothing of normalized temporal data using *locfit*. Representative images showing normalized (black line) and normalized *locfit* (red line) data in T and S strain. x-axis denotes the time-points in sporulation medium and y-axis is the log_2 expression
