# Supplementary material for: Meiotic Interactors of a Mitotic Gene TAO3 Revealed by Functional Analysis of its Rare Variant
Source: G3 (Bethesda). 2016 Jun 14;6(8):2255–63. doi: 10.1534/g3.116.029900 (PMC4978881; doi:10.1534/g3.116.029900)
Supplement: Supplemental Material [file supp_g3.116.029900_FileS1.pdf]

Meiotic interactors of a mitotic gene *TAO3* revealed by functional analysis of its rare variant

Saumya Gupta, Aparna Radhakrishnan, Rachana Nitin, Pandu Raharja-Liu, Gen Lin, Lars M. Steinmetz, Julien Gagneur, Himanshu Sinha

FILE S1
DETAILED METHODS

1. Mathematical modeling for progression through meiotic stages

Parameterization of cell stage kinetics

A multi-stage modeling was performed as described in Gupta *et al*. (2015). Cells in G_1/S phase of cell cycle were said to be in 1-nucleus state. Cells that have completed MI or MII were said to be in 2-nuclei or 4-nuclei state, respectively. Cells that did not progress from one cell cycle state to another were mentioned as inactive cells. The existence of inactive states was supported by the fact that at steady state, some cells still had 1-nucleus or 2-nuclei indicating they were trapped at these stages, which could be possibly due to nuclear destruction mechanism resulting in dyads (Eastwood *et al.* 2012). Hence cells could be either in a 1-nucleus active, 1-nucleus inactive, 2-nuclei active, 2-nuclei inactive or 4-nuclei state. Moreover the cells were assumed to only progress in one direction (no back transitions) from the 1-nucleus active to either the 1-nucleus inactive or the 2-nuclei active stage, and from the 2-nuclei active to either the 2-nuclei inactive or to the 4-nuclei state. The samples contained a large number of cells and thus we used Ordinary Differential Equations to describe the dynamics of the system. The dynamics was modeled with an initial lag phase (measured as t) followed by first order kinetics between the stages (measured as α, β, γ and d, as shown below):

$$\begin{pmatrix} X_1 \xrightarrow{a} X_2 \xrightarrow{g} X_4 \\ X_1 \xrightarrow{b} Y_1 \\ X_2 \xrightarrow{d} Y_2 \end{pmatrix}$$

where X_1 is proportion of cells in 1-nucleus active stage, X_2 in 2-nuclei active stage, X_4 in 4-nuclei active stage, Y_1 is proportion of cells in 1-nucleus inactive stage, Y_2 in 2-nucleus inactive stage. The dynamics was modeled with an initial lag phase of duration t followed by first order kinetics as follows:

$$\text{for all } t \le t : \begin{cases} X_1(t) = 1 \\ X_2(t) = X_4(t) = Y_1(t) = Y_2(t) = 0 \end{cases} \text{ and}$$

$$\text{for all } t > t : \begin{cases} \frac{dX_1}{dt} = -(a+b)X_1 \\ \frac{dX_2}{dt} = aX_1 - (g+d)X_2 \\ \frac{dX_4}{dt} = gX_2 \\ \frac{dY_1}{dt} = bX_1 \\ \frac{dY_2}{dt} = dX_2 \end{cases}$$

This model leads to the following closed form solutions:

$$\text{for all } t > \tau: \begin{cases} X_1(t) = e^{-t'(\alpha+\beta)} \\ X_2(t) = \frac{\alpha}{\mu-\lambda}\left(e^{-t'\lambda} - e^{-t'\mu}\right) \\ X_4(t) = \frac{\alpha\gamma}{\mu-\lambda}\left[\frac{1}{\lambda}\left(1-e^{-t'\lambda}\right) - \frac{1}{\mu}\left(1-e^{-t'\mu}\right)\right] \\ Y_1(t) = \frac{\beta}{\alpha+\beta}\left(1-e^{-t'(\alpha+\beta)}\right) \\ Y_2(t) = \frac{\alpha\delta}{\mu\lambda(\mu-\lambda)}\left[\lambda e^{-t'\mu} - \mu e^{-t'\lambda} + (\mu-\lambda)\right] \end{cases}$$

where $t' = t - \tau$, $\lambda = \alpha + \beta$ and $\mu = \gamma + \delta$.

Model fitting

For each background, the data consisted of frequencies $f_{i,j}$ of cells with $j \in \{1,2,4\}$ nuclei measured at time t_i. The model described above was fitted to minimize the sum of squared errors:

$$\min_{\theta} \sum_i \left(f_{i,1} - X_1(t_i) - Y_1(t_i)\right)^2 + \left(f_{i,2} - X_2(t_i) - Y_2(t_i)\right)^2 + \left(f_{i,4} - X_4(t_i)\right)^2$$

where $\theta = (\alpha,\beta,\gamma,\delta,\tau)^T$ is the vector of parameters. The cost function was minimized using the R function *optim()* with default parameters. The cost function was parameterized with the logarithm of the parameters to ensure their positivity (these were lag times and rates).

Confidence Intervals

Confidence intervals on the parameters were obtained with a leave-one-out approach, where the replicate data for each time point were left out one at a time and the model was fitted on the remaining time points. This bootstrapping scheme was more stringent and more appropriate than sampling with replacement the complete dataset without stratifying per time point because frequency measurements within one time point were closer to each other than to the fitted values. For further details see Gupta *et al.* (2015).

2. Conditional expression of *TAO3* during sporulation

A $tetO_7$-based promoter substitution cassette containing *kanMX4*, amplified from the plasmid pCM225 (Bellí *et al.* 1998), was inserted to replace the endogenous *TAO3* promoter (-150 to -1bp upstream start site) in the T strain. T strains with the endogenous promoter and the strain with $tetO_7$ promoter (P_{Tet}-*TAO3(4477C)*) were phenotyped for estimating sporulation efficiency. The strains were grown in a glucose-rich medium (YPD) and synchronized in pre-sporulation medium (YPA) prior to initiating sporulation in sporulation medium (Spo). To decrease the activity of *TAO3(4477C)* during a specific condition (YPD, YPA or Spo), doxycycline was added in the medium during that temporal phase. For instance, for decreasing the activity of *TAO3(4477C)* only during growth in glucose, P_{Tet}-*TAO3(4477C)* strain was grown in YPD with doxycycline, washed and added to YPA and Spo in the absence of doxycycline.

3. Whole genome gene-expression analysis

Time-series gene expression data was smoothed and made continuous to give a more accurate representation of the expression profile of each gene (Bar-Joseph *et al.* 2012, also see Gupta *et al.* 2015). Therefore after normalization, the $\log_2$ transformed expression values of all transcripts were made continuous over time using *locfit* (Loader 2007). This method performs local regression by relying on the bandwidth parameter '*h*'. This parameter was optimized for our expression data by controlling its value such that it gave minimum error by the leave one out method for a range of bandwidths. Briefly in leave one out method, for each strain, one time point for a single gene was 'left out' or missed and its value was predicted based on the respective '*h*' being tested. This was done for the expression values across all time points of a gene, and error was calculated between the actual and the predicted value. For optimizing '*h*' in our data, the top 10% of the transcripts ordered by the descending order of standard deviation across time in expression values were considered. For a big range of '*h*' between 1 and 20, the bandwidth with minimum error was mainly observed to be between 1 and 3. Thus this was used as the next range. In the range between 1 and 3, the most optimum bandwidth was observed to be h = 1.2. A random set of 20 transcripts were selected and plotted to select the optimum '*h*' (few shown for T and S strain in Supporting Figure S2).

A baseline transformation for each transcript, after smoothing, was done by subtracting each time point value from t = 0h (t_0), as follows:

$$y'_{S(t_n)} = y_{S(t_n)} - y_{S(t_0)}$$
$$y'_{T(t_n)} = y_{T(t_n)} - y_{T(t_0)}$$

where y is the expression value of a transcript for a strain (S or T) at a specific time point and y' is the transformed expression value. It is this $\log_2$ fold value (called as expression) that is used for all the comparisons made between M and S strains or T and S strains.

To identify differentially expressed genes (after removing tRNAs, snRNAs and transcripts from terminal repeats) between T and S strain, the temporal expression profiles of each transcript were compared using the method implemented in the EDGE (Extraction of Differential Gene Expression) software (Leek *et al.* 2006). The EDGE software requires gene expression data as input in a specific format, which was created using the R script given as follows:

```
#Create input files - data & covariate file for EDGE comparisons

data=read.table(paste(path,'TvS/TableS5.txt',sep=''), stringsAsFactors=F,
header=T, row.names=1,sep='\t')

if(file.exists("TvS/")==F) {system('mkdir TvS')}
write.table(data,quote=F,sep='\t',file="TvS/data.txt")

cov=c('Cov',colnames(data))
cov=rbind(cov,c('Strain',rep('TAO3',8),rep('S288c',8)))
time=c(30,45,70,100,150,230,340,510)
cov=rbind(cov,c('Time',rep(time,2)))
cov=rbind(cov,c('Treatment',rep(1,8),rep(0,8)))
```

```
write.table(cov, quote=F, row.names=F,
col.names=F,sep='\t',file="TvS/cov.txt")

q(save='no')
```

Optimal discovery procedure was used with 1,000 null iterations and random seed number. From EDGE analysis, we identified genes showing significant differential expression across time by keeping a 10% FDR.

The differentially expressed genes were clustered according to their temporal expression patterns using time abstraction clustering algorithm implemented in the TimeClust software (Magni *et al.* 2008). Since our data was previously smoothed, we did not smoothen it further as required by the software. The smoothened and baseline transformed expression data of the 8 sporulation time-points was analyzed with length segment parameter set at 3, to classify the expression data majorly as early middle and late. An absolute expression change of 0.1 (slope) was considered as a change. This clustering method was applied on the expression data separately for each strain.

References

Bar-Joseph Z., Gitter A., Simon I., 2012 Studying and modelling dynamic biological processes using time-series gene expression data. Nat Rev Genet 13: 552–564.

Bellí G., Garí E., Aldea M., Herrero E., 1998 Functional analysis of yeast essential genes using a promoter-substitution cassette and the tetracycline-regulatable dual expression system. Yeast 14: 1127–1138.

Eastwood M. D., Cheung S. W. T., Lee K. Y., Moffat J., Meneghini M. D., 2012 Developmentally programmed nuclear destruction during yeast gametogenesis. Dev Cell 23: 35–44.

Gupta S., Radhakrishnan A., Raharja-Liu P., Lin G., Steinmetz L. M., *et al.*, 2015 Temporal expression profiling identifies pathways mediating effect of causal variant on phenotype. PLoS Genet 11: e1005195.

Leek J. T., Monsen E., Dabney A. R., Storey J. D., 2006 EDGE: extraction and analysis of differential gene expression. Bioinformatics 22: 507–508.

Loader C., 2007 Locfit: Local regression, likelihood and density estimation. R package version.

Magni P., Ferrazzi F., Sacchi L., Bellazzi R., 2008 TimeClust: a clustering tool for gene expression time series. Bioinformatics 24: 430–432.
